# Supplementary material for: Single-organoid analysis reveals clinically relevant treatment-resistant and invasive subclones in pancreatic cancer
Source: NPJ Precis Oncol. 2023 Dec 8;7:128. doi: 10.1038/s41698-023-00480-y (PMC10709344; doi:10.1038/s41698-023-00480-y)
Supplement: Supplementary file 1 — Supplementary Information [file 41698_2023_480_MOESM1_ESM.docx]

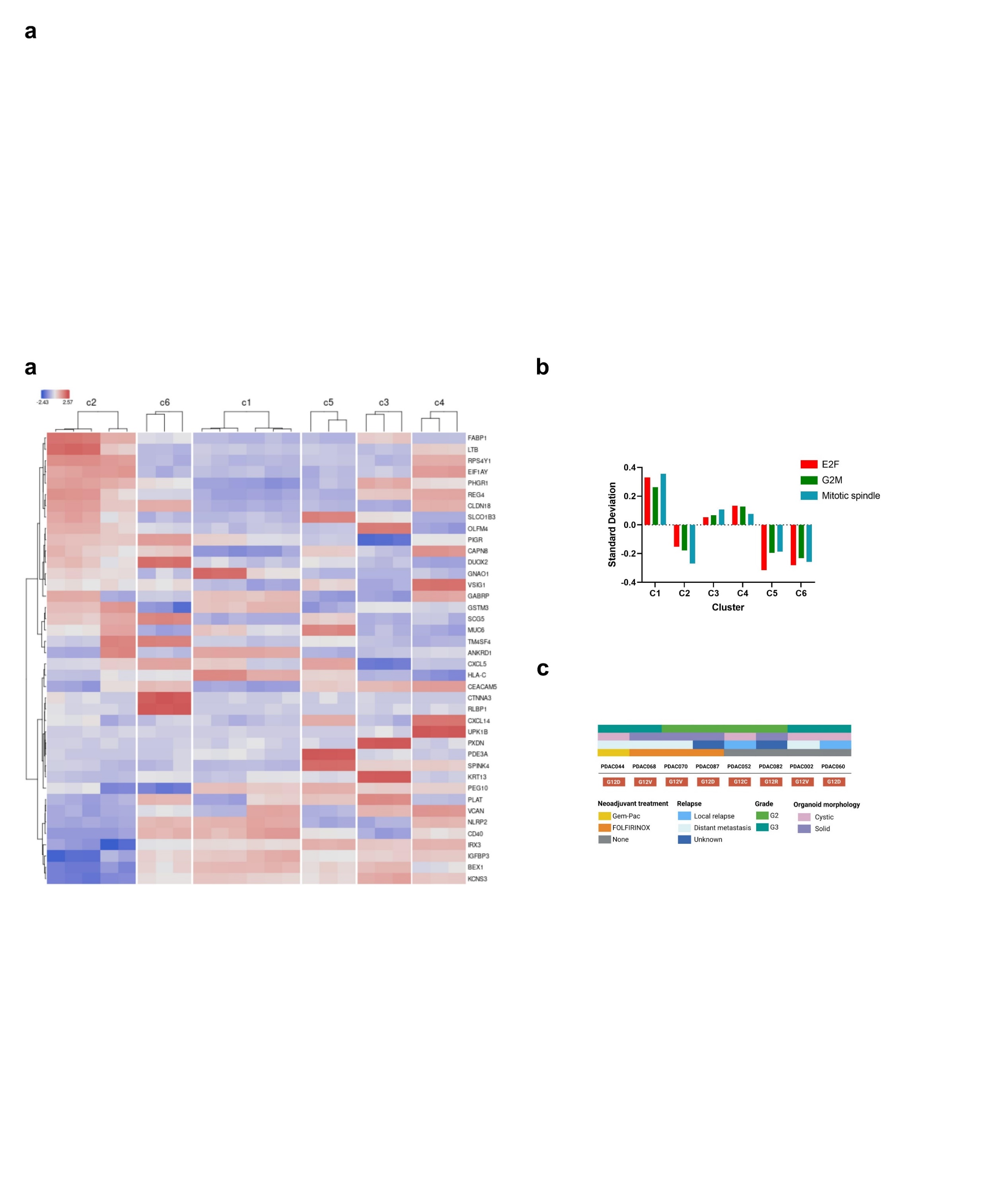


**Supplementary Figure 1. PDAC organoid cohort shows distinct molecular features. a.** Overview of the differentially expressed genes between the individual clusters (C1,C2,C3,C4,C5 and C6). **b**. Standard deviation (of the LogFC) of the selected Hallmark gene sets highlighting cluster specific growth/cell division features. **c.** Visual representation of the KRAS mutational spectrum of the included PDAC organoid cohort (N=8) with annotated clinical information (Grade, Morphology, Relapse and Neoadjuvant treatment).


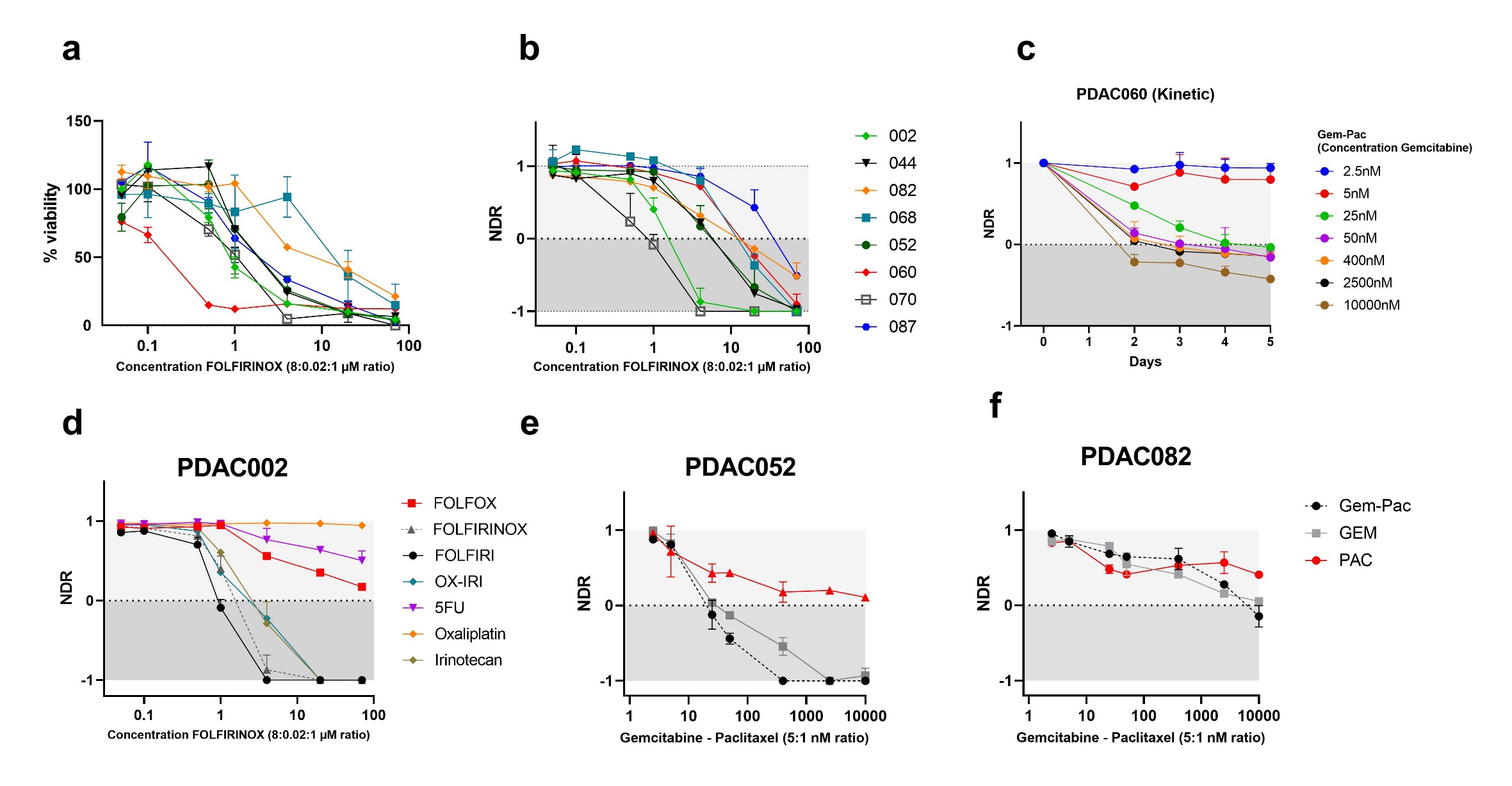


**Supplementary Figure 2. Normalized drug metric (NDR) highlights the inter-patient response heterogeneity. a-b.** % Viability and NDR dose response curve upon treatment with FOLRIRINOX (20 µM 5-FU:0.0625 µM SN38:2.5 µM Oxaliplatin ratio). **c**. Kinetic NDR quantification of PDAC060 treated with various concentrations (2.5 nM-10000 nM) gemcitabine-paclitaxel. NDR dose response curve of PDAC002 of the different FOLFIFINOX regimen. **e-f.** NDR dose response curve of PDAC052 (additive effect) and PDAC082 (no additive effect) showing the patient-specific additives effects.


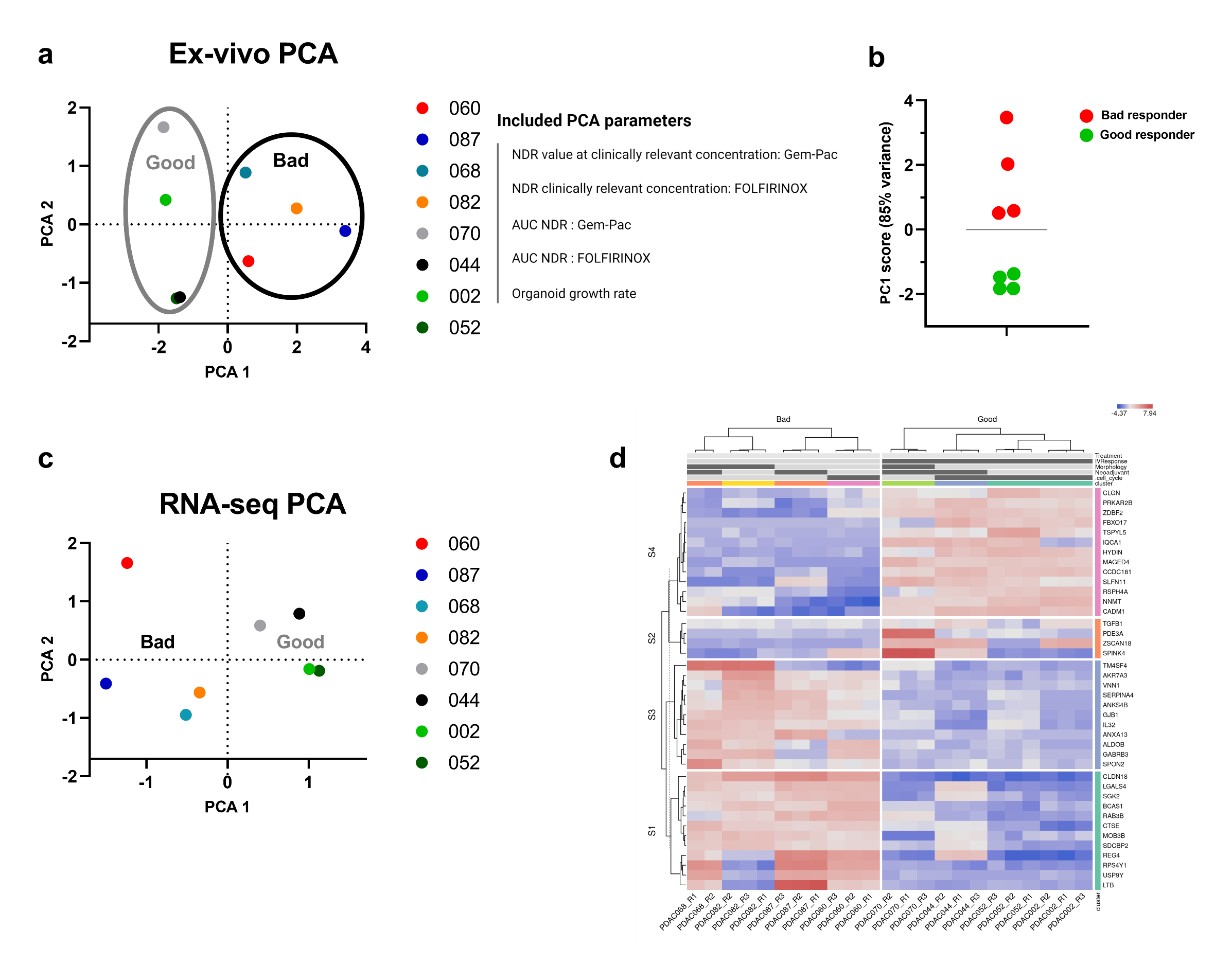


**Supplementary Figure 3. Transcriptional analysis highlights clinical relevance of NDR readout. a-c.** Principal components analyses (PCA) of either the ex-vivo based drug responses (clinically relevant concentration; 400 nM gemcitabine: 80 nM paclitaxel and 4 µM 5-FU:0.0125 µM SN38:0.5 µM Oxaliplatin) or transcriptome data (normalized read counts). **d**. Heatmap showing the top differentially expressed genes used for the biomarker identification.


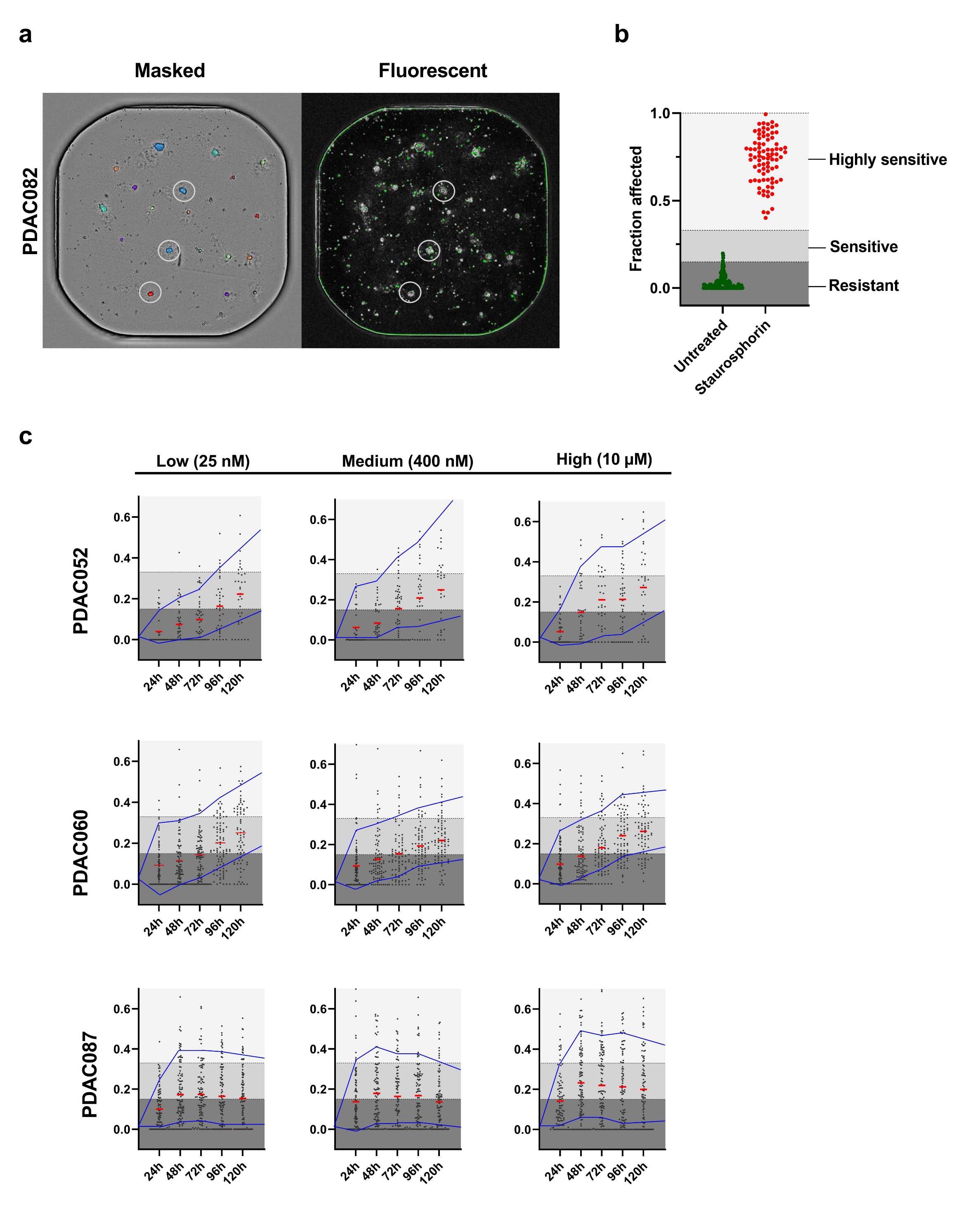


**Supplementary Figure 4. Single-organoid analysis reveals the intra-tumoral response heterogeneity. a.** Representative masked and fluorescent (cytotox green) images, showing the presence of masked PDAC organoid clones with a minor green overlap. For each masked organoids, the green overlapping area was automatically quantified (basis of the single organoid readout. **b**. Fraction affected values of the control and positive control (staurosphorine), which was used to define the response ranges (sensitive, highly sensitive and resistant). **c.** Kinetic single-organoid quantification (fraction affected) upon treatment with low (25 nM:5 nM), middle (400 nM:80 nM) or high (10000 nM and 250 nM) concentrations of gemcitabine-paclitaxel.


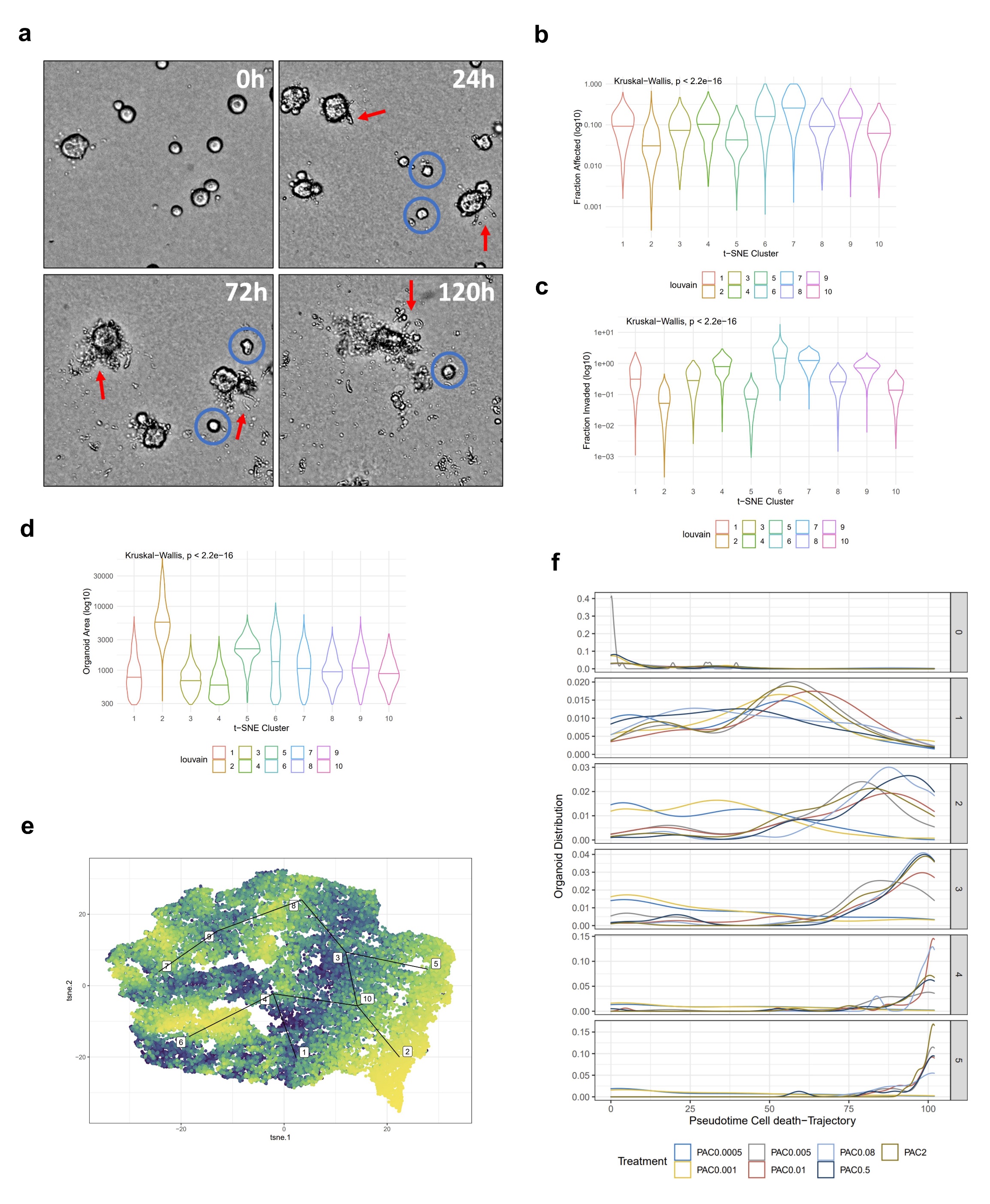


**Supplementary Figure 5. PDAC organoids reveal patient-therapy-concentration and -time specific invasive patterns. a.** Brightfield visualization of PDAC002 showing the time dependent invasive behavior. Red arrows= spindle shaped cells, Blue circles=non-invasive PDAC organoids. Scale bar=20 µm. **b-d.** Specific features (Fraction affected, Invasive fraction and organoid area) per cluster. **e.** tSNE visualization of organoids with a high area (Cluster 2 and 5). **f.** Density distribution analysis over the Fraction affected (cell death) pseudotime trajectory.
