# Supplementary material for: Single-organoid analysis reveals clinically relevant treatment-resistant and invasive subclones in pancreatic cancer
Source: NPJ Precis Oncol. 2023 Dec 8;7:128. doi: 10.1038/s41698-023-00480-y (PMC10709344; doi:10.1038/s41698-023-00480-y)
Supplement: Supplementary file 2 — Reporting summary [file 41698_2023_480_MOESM2_ESM.pdf]

Corresponding author(s): Christophe Deben
Last updated by author(s): Jun 28, 2023

Reporting Summary

Nature Portfolio wishes to improve the reproducibility of the work that we publish. This form provides structure for consistency and transparency in reporting. For further information on Nature Portfolio policies, see our Editorial Policies and the Editorial Policy Checklist.

Statistics

For all statistical analyses, confirm that the following items are present in the figure legend, table legend, main text, or Methods section.

n/a	Confirmed	
☐	☒	The exact sample size (*n*) for each experimental group/condition, given as a discrete number and unit of measurement
☐	☒	A statement on whether measurements were taken from distinct samples or whether the same sample was measured repeatedly
☐	☒	The statistical test(s) used AND whether they are one- or two-sided<br>*Only common tests should be described solely by name; describe more complex techniques in the Methods section.*
☒	☐	A description of all covariates tested
☒	☐	A description of any assumptions or corrections, such as tests of normality and adjustment for multiple comparisons
☐	☒	A full description of the statistical parameters including central tendency (e.g. means) or other basic estimates (e.g. regression coefficient) AND variation (e.g. standard deviation) or associated estimates of uncertainty (e.g. confidence intervals)
☐	☒	For null hypothesis testing, the test statistic (e.g. *F*, *t*, *r*) with confidence intervals, effect sizes, degrees of freedom and *P* value noted<br>*Give P values as exact values whenever suitable.*
☒	☐	For Bayesian analysis, information on the choice of priors and Markov chain Monte Carlo settings
☒	☐	For hierarchical and complex designs, identification of the appropriate level for tests and full reporting of outcomes
☐	☒	Estimates of effect sizes (e.g. Cohen's *d*, Pearson's *r*), indicating how they were calculated

Our web collection on statistics for biologists contains articles on many of the points above.

Software and code

Policy information about availability of computer code

Data collection	TCGA data analysis was accessed using GEPIA2 for generating survival curves.
Data analysis	AI-driven image analysis was performed by integrating the following open source software packages: h5py 2.10.0, imageio 2.9.0, opencv-python 4.5.4.58, napari 0.4.16, numpy 1.23.2, pandas 1.4.2, scikit-image 0.19.3, scikit-learn 0.24.1, scipy 1.9.1, sklearn 0.0, torch 1.10.0, torchaudio 0.10.0, torchvision 0.11.1,<br><br>NDR drug metrics, WES analysis, tSNE-plots for single organoid analysis: R-Studio (including additional packages, see manuscript).<br><br>Plots and statistical analysis: Graphad Prism 10.0.0<br><br>Transcriptome analysis: BigOmics Playground V2

For manuscripts utilizing custom algorithms or software that are central to the research but not yet described in published literature, software must be made available to editors and reviewers. We strongly encourage code deposition in a community repository (e.g. GitHub). See the Nature Portfolio guidelines for submitting code & software for further information.

Data

Policy information about availability of data

All manuscripts must include a data availability statement. This statement should provide the following information, where applicable:

- Accession codes, unique identifiers, or web links for publicly available datasets
- A description of any restrictions on data availability
- For clinical datasets or third party data, please ensure that the statement adheres to our policy

The raw drug screening datasets are available from the corresponding author on reasonable request, as it is still part of an ongoing study. The RNA datasets have been deposited in the Gene Expression Omnibus (GEO) database under accession number GSE235548.

Research involving human participants, their data, or biological material

Policy information about studies with human participants or human data. See also policy information about sex, gender (identity/presentation), and sexual orientation and race, ethnicity and racism.

Reporting on sex and gender	PDAC organoid lines derived from both Male (N=5) and Female (N=3) patients diagnosed with pancreatic cancer were included in this study. The patient information was provided by the UZA database, covered under a written informed consent.
Reporting on race, ethnicity, or other socially relevant groupings	The only socially relevant grouping mentioned in this study was gender. However, this was not taken into account for the downstream analysis.
Population characteristics	For each patient, we have obtained the following information: age, gender, initial diagnosis, stage, treatment response.
Recruitment	Patients were not actively recruited for this study, but rather selected derived organoid lines based on predefined characteristics (see manuscript).
Ethics oversight	Human specimens and clinical data were obtained from the Antwerp University hospital under approval by the Ethical Committee for Medical Ethics UAntwerp/UZA on 17/11/2020 with reference number 14/47/480.

Note that full information on the approval of the study protocol must also be provided in the manuscript.

Field-specific reporting

Please select the one below that is the best fit for your research. If you are not sure, read the appropriate sections before making your selection.

☒ Life sciences ☐ Behavioural & social sciences ☐ Ecological, evolutionary & environmental sciences

For a reference copy of the document with all sections, see nature.com/documents/nr-reporting-summary-flat.pdf

Life sciences study design

All studies must disclose on these points even when the disclosure is negative.

Sample size	A panel of 8 PDAC organoid lines was selected based on their discriminative features (e.g. clinical response, morphology). The amount of included patients is in accordance with other proof of concept studies.
Data exclusions	No data was excluded.
Replication	The image analysis and assay workflow have been comprehensively validated (performance, reproducibility) in two previous manuscripts (Deben et al. Cell Oncol 2023 and Le Compte et al. JOVE 2023).
Randomization	Since the aim of this study was to identify patient-specific differences, we did not included a randomization step (also due to the small sample size of N=8)
Blinding	Blinding was not relevant for this study since all the data was included in the manuscript. Moreover there was no data that could influence the outcome.

Reporting for specific materials, systems and methods

We require information from authors about some types of materials, experimental systems and methods used in many studies. Here, indicate whether each material, system or method listed is relevant to your study. If you are not sure if a list item applies to your research, read the appropriate section before selecting a response.

Materials & experimental systems

n/a	Involved in the study
☒	☐ Antibodies
☐	☒ Eukaryotic cell lines
☒	☐ Palaeontology and archaeology
☒	☐ Animals and other organisms
☒	☐ Clinical data
☒	☐ Dual use research of concern
☒	☐ Plants

Methods

n/a	Involved in the study
☒	☐ ChIP-seq
☒	☐ Flow cytometry
☒	☐ MRI-based neuroimaging

Eukaryotic cell lines

Policy information about cell lines and Sex and Gender in Research

Cell line source(s)	Primary pancreatic tumor organoid organoid lines were derived from PDAC patients (female N=3 and male N=5).
Authentication	Whole exome sequencing was performed to identify KRAS mutations as a marker for malignancy.
Mycoplasma contamination	All PDAC organoid lines have tested negative on mycoplasma contamination.
Commonly misidentified lines (See ICLAC register)	/
